# Intravenous Immunoglobulin Treatment in Patients With Streptococcal Toxic Shock Syndrome in Southern Sweden: A Retrospective Population-Based Study

**DOI:** 10.1093/ofid/ofag092

**Published:** 2026-02-28

**Authors:** Olof Wullt, Charlotta Utbult, Erik Carlson, Oskar Ljungquist, Torgny Sunnerhagen, Anna Bläckberg, Gustav Torisson

**Affiliations:** Department of Infectious Diseases, Skåne University Hospital, Malmö, Sweden; Department of Infectious Diseases, Helsingborg Hospital, Helsingborg, Sweden; Department of Infectious Diseases, Skåne University Hospital, Malmö, Sweden; Department of Infectious Diseases, Helsingborg Hospital, Helsingborg, Sweden; Infection Medicine, Department of Clinical Sciences, Lund University, Helsingborg, Sweden; Department of Clinical Microbiology, Skåne University Hospital, Lund, Sweden; Infection Medicine, Department of Clinical Sciences, Lund University, Lund, Sweden; Infection Medicine, Department of Clinical Sciences, Lund University, Lund, Sweden; Department of Infectious Diseases, Skåne University Hospital, Lund, Sweden; Department of Infectious Diseases, Skåne University Hospital, Malmö, Sweden; Clinical Infection Medicine, Department of Translational Medicine, Lund University, Malmö, Sweden

**Keywords:** intravenous immunoglobulins, septic shock, streptococcal toxic shock syndrome

## Abstract

**Background:**

Intravenous immunoglobulins (IVIGs) have been suggested as an adjunctive treatment in streptococcal toxic shock syndrome (STSS), but there are no conclusive trials. In southern Sweden, IVIG is routinely used in certain hospitals but not others. We hypothesized that this would resemble a natural experiment, and we aimed to evaluate the effect of IVIG in patients with STSS.

**Method:**

We conducted a population-based retrospective cohort study on STSS cases in southern Sweden from 2017 to 2024. The main exposure was any IVIG treatment, and the primary outcome was 30-day mortality. Cox regression was used, adjusted for lactate, Sequential Organ Failure Assessment score, Charlson Comorbidity Index, and concurrent clindamycin treatment. We modeled IVIG as a time-dependent variable to address immortal time bias.

**Results:**

In total, 106 patients fulfilled STSS criteria, of which 56 (53%) were treated with IVIG. Despite geographic differences, the IVIG group was younger and had fewer comorbidities but higher disease severity at baseline. Crude analysis suggested lower mortality in the IVIG group (hazard ratio, 0.69; 95% CI, .34–1.41). However, after adjusting for covariates and accounting for immortal time bias, the hazard ratio was estimated at 1.69 (95% CI, .66–4.30).

**Conclusions:**

Although our study included a large population of patients with STSS, our results were inconclusive regarding the effect of IVIG on 30-day mortality. This study highlights the risk of bias in observational studies in rare conditions. Prospective interventional studies are needed to determine the efficacy of IVIG in patients with STSS.

## Teaser

In this population-based study of 106 cases with streptococcal toxic shock syndrome, our results were inconclusive regarding the effect of intravenous immunoglobulin on 30-day mortality. Cautious interpretation is warranted due to the risk of remaining bias. Prospective studies are needed.

## Graphical Abstract

**Graphical Abstract:**
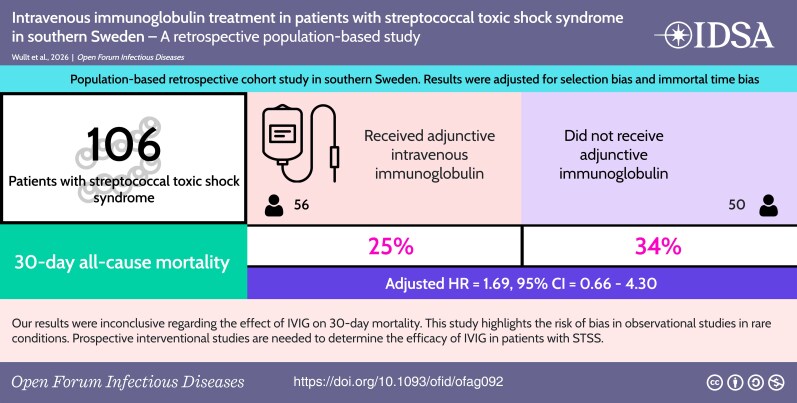
This graphical abstract is also available at Tidbit: https://tidbitapp.io/tidbits/intravenous-immunoglobulin-treatment-in-patients-with-streptococcal-toxic-shock-syndrome-in-southern-sweden-a-retrospective-population-based-study-b705d7fd-0403-4929-a5db-5625b763cfe4

The incidence of invasive group A streptococcus (iGAS) infections has increased in several locations after the COVID-19 pandemic [1]. One of the most severe forms of iGAS is streptococcal toxic shock syndrome (STSS), occurring in 10% to 15% of iGAS cases, with short-term mortality ranging from 23% to 44% [2, 3]. Treatment of STSS includes β-lactam antibiotics as well as adjunctive clindamycin, which has been shown to inhibit bacterial toxin production and improve outcomes [2, 4, 5]. In addition, surgery is often necessary for source control and supportive care, predominantly in the intensive care setting [3, 6].

The survival benefit of adjunctive intravenous immunoglobulin (IVIG) treatment in STSS is debated. Several mechanistic studies have provided biological plausibility for a positive effect [7, 8]. However, clinical studies are limited to 1 prematurely stopped randomized controlled trial and observational studies, with somewhat divergent results [4, 9–17]. A meta-analysis of the crude effect in clindamycin-treated cases from 5 studies, with a population of 165 patients, described a pooled mortality of 16% in the IVIG group, as compared with 34% in the non-IVIG group, suggesting a beneficial effect of IVIG in clindamycin-treated STSS, although the authors acknowledge the risk of confounding and bias [15].

In southern Sweden, the shortage of conclusive clinical studies has led to different interpretations of the current evidence. Consequently, treatment traditions differ among hospitals in the region. In the 2 major university hospitals in Lund and Malmö, adjunctive IVIG is administered routinely in one but not the other. We hypothesized that this would represent a natural experiment, in which the treatment would depend primarily on the site of residence, thus reducing selection bias.

Therefore, we aimed to carry out a population-based study in this region to determine the effect of adjunctive IVIG treatment on 30-day mortality in patients with STSS.

## METHODS AND MATERIALS

This retrospective population-based cohort study was conducted in the Skåne region in southern Sweden, with 1.4 million inhabitants. The region is served by 10 hospitals: the 2 university hospitals in Malmö and Lund, 3 midsize hospitals with intensive care units (ICUs; Helsingborg, Kristianstad, and Ystad), and 5 community hospitals without intensive care capacities. Any patient requiring intensive care presenting to a community hospital will be transferred to a hospital with ICU capacity. In the region, all aspects of modern health care are provided except liver transplantation and intensive care for patients with burns. All hospitals in the region are served by the Department of Clinical Microbiology in Lund, with a comprehensive microbiological database. Data were collected through manual review of electronic medical records, as performed by resident physicians (C. U., O. W., E. C.) according to a prespecified protocol and supervised by an infectious disease consultant (G. T.). The primary outcome was 30-day mortality. The study was approved by the Swedish Ethical Review Authority.

### Case Finding of iGAS in the Microbiology Database

For case finding, data were retrieved from the Department of Clinical Microbiology in Lund, including cultures with group A streptococcus (GAS) obtained from blood or another normally sterile site (deep tissue biopsy, synovial fluid, cerebrospinal fluid, pericardial fluid, pleural fluid, or another sterile site) between 1 January 2017 and 31 December 2024 in a resident of Skåne region. An episode was excluded if the patient had no symptom of infection at the time of culture or if symptoms could not be determined due to the medical record being unavailable. Patients with GAS in a sterile site and symptoms were considered to have iGAS infection. An individual could be included multiple times in the study if that patient had 2 separate iGAS episodes. To separate iGAS episodes, we required that ≥30 days had elapsed since the previous episode and the patient had been discharged from the hospital in the interim. Baseline was defined as the time point when the iGAS-defining culture had been obtained. Microbiological data included *emm* types when available.

### Definition of STSS Cases

The final study population consisted of patients with STSS, defined in accordance with the definition used by the Working Group on Severe Streptococcal Infections [18]. To fulfill STSS criteria, verified iGAS and hypotension (≤90 mm Hg, systolic blood pressure) were required, with at least 2 of the following: acute respiratory distress syndrome, liver involvement, generalized erythematous rash, coagulopathy, soft tissue necrosis, or renal impairment. Any missing values in STSS criteria (eg, rash was not mentioned in medical record) were considered normal or absent.

### Baseline Characteristics

Demographic data were collected, including age, sex, and functional status (if patients were receiving municipal home care or lived in a nursing home). Patients’ preexisting disease burden was evaluated by the Charlson Comorbidity Index (CCI), including age [19]. Other comorbidity data included skin disease predisposing for infection and current immunosuppressive treatment. Ceiling of care status was collected, defined as a doctor's documented recommendation not to escalate to ICU.

### Clinical Presentation

We recorded the following symptoms: fever, pulmonary symptoms, gastrointestinal symptoms, localized pain, erythema, symptoms from ear/pharynx, or other (eg, headache). Laboratory values and vital signs at baseline included plasma lactate, platelet count, bilirubin, and creatinine. Vital signs were collected according to the National Early Warning Score 2 (NEWS2), per local routine [20]. We also estimated the Sequential Organ Failure Assessment (SOFA) score at baseline [21]. Occurrence of septic shock was assessed per the Sepsis-3 definition [22].

### Interventions

IVIG exposure was defined as having received at least 1 dose of IVIG with the purpose of treating STSS. The number of doses, total accumulated IVIG dose, and time from baseline to first IVIG administration were collected. Normally, IVIG would be administrated in a dose of 1 g/kg (maximum, 50 g) on day 1, with subsequent doses day 2 and 3 if the patient is still in shock. Other in-hospital interventions and treatments included choice of β-lactam antibiotic and whether adjunctive clindamycin was given. In addition, surgery, ICU care, ventilator treatment, renal replacement therapy, and vasopressor treatment were recorded. Infection focus was determined subjectively by the reviewer at the end of each chart review, incorporating symptoms, culture findings from sterile and nonsterile sites, radiologic findings, *ICD* codes from surgery notes, and discharge. Outcomes included 30-day mortality (primary outcome) as well as 7-day mortality, in-hospital mortality, 90-day mortality, and length of stay for those surviving to discharge.

### Statistical Analysis

All variables in the IVIG and non-IVIG groups were presented as median (IQR) or count with percentage. Groups were compared with Fisher exact tests, χ^2^ tests, and Mann-Whitney tests, as appropriate. In case of missing values, we used complete case analysis for the specific variables. In the estimation of composite scores such as CCI, SOFA, or NEWS2, missing values in the different subitems were assumed to be normal; for example, if bilirubin had not been obtained, we considered this to be normal, resulting in 0 points for this subitem when calculating the SOFA score.

The crude effect on the risk for 30-day mortality was estimated by a Cox proportional hazards model and presented as a hazard ratio (HR) with 95% CIs. The exposure was coded as 0 = no IVIG and 1 = IVIG and the outcome as 0 = alive and 1 = deceased. Thus, an HR <1 would be in favor of IVIG. To address imbalances between the IVIG and non-IVIG groups, adjustments were made for disease severity (plasma lactate, SOFA score), comorbidities (CCI with age), and adjunctive clindamycin treatment. A priori selection of these variables was based on previous research and their clinical relevance. A time-to-event model was chosen to address immortal time bias, with IVIG as a time-dependent variable. In this analysis, patients who received IVIG belonged to the non-IVIG group until the first dose of IVIG was given, after which they were allocated to the IVIG group. To visualize the effect and direction of selection bias, these adjustments were performed stepwise in the Cox proportional hazards model, with each variable added individually and plotted in a forest plot. To determine which other factors were associated with mortality, we performed an exploratory analysis with univariate Cox regression models for each variable vs the primary outcome. All analyses were performed in R, and the code is found at www.github.com/gtorisson/iGAS.

## RESULTS

**Figure 1.**
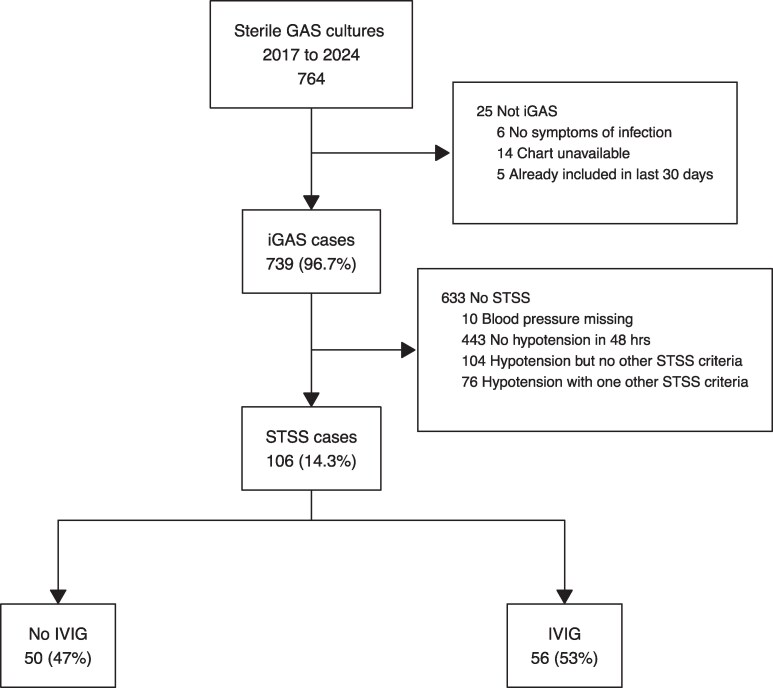
Flowchart describing the identification of patients with STSS. GAS, group A streptococcus; iGAS, invasive group A streptococcus; IVIG, intravenous immunoglobulin; STSS, streptococcal toxic shock syndrome.

In total, 764 episodes with GAS obtained from a sterile site were identified. Of these episodes, 25 (3.3%) were excluded for a lack of symptoms (n = 6), unavailable charts (n = 14), and previous inclusion in the preceding 30 days (n = 5). The remaining 739 episodes were considered iGAS episodes, occurring in 734 individuals, with 5 persons having 2 iGAS episodes during the 8-year follow-up. In total, 106 episodes (14.3%) in 106 unique patients fulfilled STSS criteria upon review, with 56 subsequently receiving IVIG and 50 not (Figure 1). As hypothesized, the proportion of patients with STSS receiving IVIG differed among hospitals in the region: in Malmö, 3 of 27 (11%) received IVIG; in Lund, 23 of 31 (74%); and in Helsingborg, 21 of 31 (68%).

The median age was 62 years, with IVIG recipients being younger than those who did not receive IVIG. Most comorbidities were less frequent in the IVIG group, including heart disease, diabetes, and malignancies. The group receiving IVIG also had a lower Charlson comorbidity score and were less likely to have home care or a ceiling of care decision (Table 1).

**Table 1.** Patient Demographics and Comorbidities

	No IVIG (n = 50)	IVIG (n = 56)	*P* Value
Age, y	65 (52–79)	60 (49–72)	.17
Male sex	30 (60)	40 (71)	.21
Hospital city			<.001
Malmö	24 (48)	3 (5.4)	
Lund	8 (16)	23 (41)	
Helsingborg	8 (16)	21 (38)	
Kristianstad	5 (10)	9 (16)	
Other	5 (10)	0 (0)	
Comorbidities			
Ischemic heart disease	9 (18)	4 (7.1)	.089
Heart failure	9 (18)	0 (0)	<.001
Cerebrovascular disease	3 (6.0)	3 (5.4)	>.99
Chronic pulmonary disease	4 (8.0)	4 (7.1)	>.99
Connective tissue disease	5 (10)	6 (11)	.90
Chronic hepatic disease	2 (4.0)	3 (5.4)	>.99
Diabetes mellitus	15 (30)	4 (7.1)	.002
Active malignancy	6 (12)	3 (5.4)	.30
Chronic skin disorder	11 (22)	5 (8.9)	.061
Immunosuppression	8 (16)	6 (11)	.42
Any chronic comorbidity	31 (62)	19 (34)	.004
Home care or nursing home	8 (16)	2 (3.6)	.04
Ceiling of care	6 (12)	2 (3.6)	.14
Charlson Comorbidity Index	3 (1–5)	2 (0–3)	.022

All values are expressed as median (Q1–Q3) or No. (%).

Abbreviation: IVIG, intravenous immunoglobulin.

Symptoms were similar across groups, with fever and gastrointestinal symptoms being the most common, occurring in approximately 70% and 50%, respectively. Disease severity was higher in the IVIG group, as reflected by lower blood pressure, higher NEWS2 and SOFA scores, and higher plasma lactate. All STSS criteria were more common in the IVIG group (Table 2).

**Table 2.** Clinical Presentation at Baseline

Clinical Presentation	No IVIG (n = 50)	IVIG (n = 56)	*P* Value
Symptoms			
Fever	36 (72)	40 (71)	.95
Gastrointestinal	24 (48)	28 (50)	.84
Localized pain	14 (28)	23 (41)	.16
Dyspnea	12 (24)	20 (36)	.19
ENT symptoms	5 (10)	5 (8.9)	>.99
Erythema	12 (24)	7 (13)	.12
Other symptom	7 (14)	6 (11)	.61
Disease severity			
Systolic blood pressure, mm Hg	100 (85–120)	90 (80–120)	.38
Heart rate per minute	110 (100–124)	112 (100–134)	.34
Respiratory rate per minute	30 (20–36)	32 (28–40)	.024
Mental alteration	8 (16)	14 (25)	.23
NEWS2 score	8 (6–11)	10 (8–12)	.005
SOFA score	7.5 (5–10)	10 (8–11)	<.001
Lactate, mmol/L	4.3 (3.2–7.2)	6.8 (4.9–9.4)	<.001
Septic shock	42 (84)	53 (95)	.073
STSS criteria			
Necrosis	15 (30)	25 (45)	.12
ARDS	14 (28)	20 (36)	.40
Rash	5 (10)	19 (34)	.003
Acute renal failure	37 (74)	44 (79)	.58
Coagulopathy	26 (52)	37 (66)	.14
Liver involvement	24 (48)	36 (64)	.091

Values are presented as median (Q1–Q3) or No. (%).

Abbreviations: ARDS, acute respiratory distress syndrome; ENT, ear/nose/throat; IVIG, intravenous immunoglobulin; NEWS2, National Early Warning Score 2; SOFA, Sequential Organ Failure Assessment; STSS, streptococcal toxic shock syndrome.

The most frequent *emm* type was *emm* 1 in both groups, occurring in 57 of 106 (54%), slightly more common in the IVIG group. The most common source of infection was soft tissue infections, in 55 of 106 (52%) patients. Infection foci were quite similarly distributed between the groups. A positive blood culture was obtained in 99 (93%) episodes. In patients receiving IVIG, a larger proportion had GAS in other sterile sites as well (Table 3).

**Table 3.** Microbiology and Infection Focus

	No IVIG (n = 50)	IVIG (n = 56)
*emm* type		
1	22 (47)	35 (65)
89	8 (17)	4 (7.4)
4	2 (4.3)	6 (11)
12	5 (11)	1 (1.9)
28	3 (6.4)	1 (1.9)
22	0 (0)	3 (5.6)
3	2 (4.3)	0 (0)
77	1 (2.1)	1 (1.9)
6	1 (2.1)	0 (0)
81	1 (2.1)	0 (0)
119	1 (2.1)	0 (0)
9	0 (0)	1 (1.9)
44	0 (0)	1 (1.9)
118	1 (2.1)	0 (0)
87	0 (0)	1 (1.9)
Infection focus		
Soft tissue	25 (50)	30 (54)
Pulmonary	11 (22)	8 (14)
Bacteremia with unknown focus	7 (14)	11 (20)
Other	6 (12)	2 (3.6)
ENT focus	1 (2.0)	5 (8.9)
Positive blood culture	48 (96)	51 (91)
GAS in another sterile site	12 (24)	25 (45)
GAS in nonsterile site	31 (62)	31 (55)

Values are presented as No. (%).

Abbreviations: ENT, ear/nose/throat; GAS, group A streptococcus; IVIG, intravenous immunoglobulin.

In IVIG recipients, the median time from baseline to the first IVIG dose was 10 hours. Of 56 patients, 30 received a single IVIG dose, 10 two doses, and 15 three doses (in 1 case, the number of doses could not be determined). The median accumulated dose equaled 0.8 g/kg. Patients in the IVIG group received empiric carbapenems to a greater extent as well as adjunctive clindamycin treatment. All other interventions, including surgery, were also more common in the IVIG group (Table 4).

**Table 4.** Interventions and Outcomes

Interventions and Outcomes	No IVIG (n = 50)	IVIG (n = 56)	*P* Value
Antibiotics			
Empiric β-lactam			.089
Benzylpenicillin	2 (4.0)	1 (1.8)	
Cloxacillin	1 (2.0)	0 (0)	
Cefotaxime	29 (58)	22 (39)	
Piperacillin/tazobactam	7 (14)	9 (16)	
Carbapenem	11 (22)	24 (43)	
Adjunctive clindamycin	36 (72)	56 (100)	<.001
Other interventions			
Intensive or intermediate care unit	43 (86)	56 (100)	.004
Surgical intervention	21 (42)	39 (70)	.004
Mechanical ventilation or CPAP	27 (54)	45 (80)	.004
Renal replacement therapy	8 (16)	27 (48)	<.001
Vasopressor therapy	42 (84)	55 (98)	.012
IVIG			
Time to IVIG, h	…	10 (4–25)	…
No. of IVIG doses			…
1	…	30 (55)	
2	…	10 (18)	
3	…	15 (27)	
Total IVIG dose, g/kg	…	0.80 (0.57–1.08)	…
Outcomes			
Length of stay, d	16 (12–24)	32 (14–45)	.010
In-hospital mortality	17 (34)	15 (27)	.42
7-d mortality	17 (34)	11 (20)	.094
30-d mortality	17 (34)	14 (25)	.28
90-d mortality	17 (34)	15 (27)	.38

Values are presented as median (IQR) or No. (%).

Abbreviations: CPAP, continuous positive airway pressure; IVIG, intravenous immunoglobulin.

The primary outcome (30-day mortality) occurred in 31 of 106 (29%) patients. The median time to death was 27 hours (IQR, 18–73) from baseline. In the non-IVIG group, all patients died within the first 7 days. The 30-day mortality rate was 14 of 56 (25%) in the IVIG group and 17 of 50 (34%) in the non-IVIG group, resulting in a crude HR of 0.69 (95% CI, .34–1.41). When adjustments were made for CCI and clindamycin treatment, the HR increased. When adjustments for lactate and SOFA were included, the HR decreased. Finally, when the model was adjusted for immortal time bias and all covariates, the HR for IVIG was 1.69 (.66–4.30; Table 5, Supplementary Figure 1).

**Table 5.** Hazard Ratios for Different Regression Models for the Effect of IVIG vs 30-Day Mortality

Model	HR	95% CI	*P* Value
Crude	0.69	.34–1.41	.31
Adjusted for SOFA and lactate	0.50	.23–1.08	.08
Adjusted for CCI and clindamycin	1.01	.44–2.30	.98
Time dependent: crude model	1.18	.57–2.44	.66
Final model: adjusted for all	1.69	.66–4.30	.27

Crude models show only the estimate for IVIG vs mortality. IVIG and mortality are coded as 0 = no and 1 = yes; thus, an HR <1 indicates lower mortality in the IVIG group. In the final model, IVIG was modeled as a time-dependent covariate, and the model was adjusted for SOFA, lactate, CCI, and clindamycin.

Abbreviations: CCI, Charlson Comorbidity Index; HR, hazard ratio; IVIG, intravenous immunoglobulin; SOFA, Sequential Organ Failure Assessment.

In the exploratory univariate analysis of other factors associated with outcome, age, comorbidities, and functional dependency were more strongly associated with mortality than markers of acute disease severity (Supplementary Tables 1–3).

## DISCUSSION

In this population-based observational study, we found no clear evidence of an effect of IVIG treatment on 30-day mortality in patients with STSS. Our hypothesis of a natural experiment was disproved, as there were substantial differences between the groups, which complicated the interpretation of our results.

As hypothesized, a greater proportion of patients with STSS in Lund and Helsingborg received IVIG as compared with Malmö. Despite this, we observed considerable between-group differences: patients who received IVIG were younger and less frail, with fewer chronic diseases and less functional impairment. There were also clear indications of higher acute disease severity in the IVIG group, with greater NEWS2 and SOFA scores, as well as increased plasma lactate. Patients receiving IVIG were more likely to receive other interventions, including surgery and adjunctive clindamycin treatment. These group differences resemble those found in previous observational studies, where patients receiving IVIG were younger, had fewer comorbidities, and were treated with adjunctive clindamycin to a larger extent [4, 9, 10].

The crude HR estimate of 0.69 for IVIG vs 30-day mortality signaled that receiving IVIG was associated with a 31% decrease in crude mortality. However, the aforementioned group differences suggest selection bias. The higher disease severity in patients receiving IVIG is likely to have increased the apparent mortality in this group. Consequentially, adjusting for lactate levels and SOFA score, the HR decreased from 0.69 to 0.50. Correspondingly, the younger age, lower degree of comorbidity, and higher frequency of adjunctive clindamycin treatment may have decreased the apparent mortality in the IVIG group. Subsequently, upon adjusting for CCI and clindamycin treatment, the HR increased from 0.69 to 1.01. Table 5 and Supplementary Figure 1 illustrate how different adjustments with regard to selection bias may lead to different results. With small sample sizes, composite clinical scores reduce the number of adjustment variables, mitigating the risk of overfitting. We used CCI and SOFA scores to enable separation of chronic comorbidity and acute disease severity. Previous studies have used scoring based on APACHE II (Acute Physiology and Chronic Health Evaluation II) [9, 23] or SAPS II (Simplified Acute Physiology Score) [10, 16, 24]. These scores combine markers for disease severity and comorbidities but are tilted toward measuring current disease severity. Therefore, adjusting for these would only to a lesser degree adjust for differences in comorbidities, which could retain bias. This was supported by our exploratory analysis, in which chronic conditions appeared more strongly associated with mortality than acute disease severity.

Observational studies evaluating an intervention occurring after baseline also risk being subjected to immortal time bias [25, 26]. This risk becomes pronounced when the outcome occurs soon after baseline and the intervention is delayed. This might be the case if the administration of IVIG is withheld until a definitive microbiological diagnosis is made. In our patients, mortality occurred early, at a median 27 hours after baseline. Immortal time bias may have favored IVIG treatment in the crude analysis, as these patients would by definition have survived the initial critical period before receiving IVIG. However, treatment was not extensively delayed, with a median time to IVIG of 10 hours, indicating that many patients received IVIG before a definitive microbiological diagnosis. When IVIG was modeled as a time-dependent covariate, the HR increased from 0.69 to 1.18, suggesting that immortal time bias may have influenced crude results. Yet, the exact reason for initiating IVIG treatment at that time point was not known. If IVIG was used as a “last resort” due to further clinical deterioration from baseline, time-dependent modeling may also introduce bias, disfavoring IVIG if given too late [27].

Strengths of this study include a population-based cohort that is characterized in large detail, including exact times for IVIG distribution and outcome. This is the first observational study on adjunctive IVIG in STSS attempting to address immortal time bias, with a larger study population than most studies on this subject [4, 9–12, 14, 17]. However, despite covering >10 million person-years of follow-up, only 106 STSS cases were included. Although certain aspects of the hypothesis of a natural experiment were correct, it failed in creating a balance in covariates between the treatment groups, which is a major limitation. In our most comprehensive model, we included 5 covariates despite having only 31 events, suggesting overfitting; this precluded further adjustments, such as choice of β-lactam, which may have influenced results. The accumulated dose of IVIG was lower than in many recommendations, which may have affected the results. Data on the reason to choose a specific dosage regime were not available, but the presence of acute respiratory distress syndrome or acute kidney failure could have led to lowered doses. Furthermore, although the medical records were generally well documented, some records were incomplete. The representativity of our study population could be questioned: applying STSS criteria retrospectively will most likely identify a population slightly different from that in a prospective setting. Nevertheless, 90% of cases fulfilled septic shock criteria, and the proportion of iGAS cases fulfilling STSS criteria and mortality rates were similar to previous studies [3].

This study highlights the challenges with observational studies involving rare conditions. Small sample sizes and unbalanced groups limit the possibilities to adjust for confounders, reducing the ability to draw conclusions. A lack of power is also an issue, with clinically relevant differences being statistically insignificant. Our result regarding immortal time bias further signals that the interpretation of observational studies is intricate. We conclude that a randomized controlled trial is required to determine if adjunctive IVIG is beneficial or not in STSS. Such a study would preferably randomize patients early, before a definitive microbiological diagnosis, accepting the risk of administering IVIG to patients not having STSS. Owing to the rarity of STSS, such an randomized controlled trial must be an international collaboration to reach statistical power.

## CONCLUSION

Although our study included a large population of patients with STSS, our results were inconclusive regarding the effect of IVIG on 30-day mortality. This study highlights the risk of bias in observational studies in rare conditions. Prospective interventional studies are needed to determine the efficacy of IVIG in patients with STSS.

## Supplementary Material

ofag092_Supplementary_Data

## Supplementary Data

Supplementary materials are available at *Open Forum Infectious Diseases* online. Consisting of data provided by the authors to benefit the reader, the posted materials are not copyedited and are the sole responsibility of the authors, so questions or comments should be addressed to the corresponding author.
