# Supplementary material for: Intravenous Immunoglobulin Treatment in Patients With Streptococcal Toxic Shock Syndrome in Southern Sweden: A Retrospective Population-Based Study
Source: Open Forum Infect Dis. 2026 Feb 28;13(3):ofag092. doi: 10.1093/ofid/ofag092 (PMC12994474; doi:10.1093/ofid/ofag092)
Supplement: ofag092_Supplementary_Data [file ofag092_supplementary_data.docx]

**SUPPLEMENTARY APPENDIX**

**Intravenous immunoglobulin treatment in patients with streptococcal toxic shock syndrome in southern Sweden –** A retrospective population-based study

Olof Wullt, Charlotta Utbult, Erik Carlson, Oskar Ljungquist, Torgny Sunnerhagen, Anna Bläckberg, Gustav Torisson

**Figure S1. Forest plot displaying the effect of different adjustment models for IVIG vs 30-day mortality.** Left-hand panel indicates the selected adjustment variables. Middle panel; direction and magnitude of impact on apparent benefit of IVIG. Right-hand panel; hazard ratios (HR), 95% confidence intervals (CI) and p-values. Time-dependent analyses are adjusted for immortal time bias. IVIG = Intravenous immunoglobulins, SOFA = Sequential Organ Failure Assessment, CCI = Charlson Comorbidity Index.

**Table S1 to S3. Exploratory univariate analysis vs mortality**

These tables show the associations between all variables and 30-day mortality using Cox regression models. The results should be interpreted carefully as there are many confounders, risk for selection bias and multiple comparisons.

| Variable | Alive N = 75 | Deceased N = 31 | HR (95% CI) | P value |
| --- | --- | --- | --- | --- |
| Age | 57 (48-71) | 72 (57-79) | 1.02 (1.00 – 1.05) | **0.034** |
| Male sex | 53 (71%) | 17 (55%) | 0.5 (0.3 – 1.1) | 0.094 |
| **Hospital city** |  |  |  |  |
| Malmö | 21 (28%) | 6 (19%) | Reference | NA |
| Lund | 21 (28%) | 10 (32%) | 1.5 (0.5 – 4.1) | 0.44 |
| Helsingborg | 23 (31%) | 6 (19%) | 0.9 (0.3 – 2.7) | 0.83 |
| Kristianstad | 9 (12%) | 5 (16%) | 1.7 (0.5 – 5.5) | 0.400 |
| Other | 1 (1%) | 4 (13%) | 3.9 (1.1 – 14.0) | **0.035** |
| Ischemic heart disease | 5 (6.7%) | 8 (26%) | 3.0 (1.3 – 6.6) | **0.008** |
| Heart failure | 5 (6.7%) | 4 (13%) | 1.7 (0.6 – 4.8) | 0.33 |
| Cerebrovascular disease | 2 (2.7%) | 4 (13%) | 4.2 (1.5 – 12.0) | **0.008** |
| Chronic pulmonary disease | 6 (8.0%) | 2 (6.5%) | 0.8 (0.2 – 3.2) | 0.72 |
| Connective tissue disease | 2 (2.7%) | 9 (29%) | 6.3 (2.9 – 13.8) | **<0.001** |
| Chronic hepatic disease | 3 (4.0%) | 2 (6.5%) | 1.5 (0.4 – 6.3) | 0.58 |
| Diabetes mellitus | 12 (16%) | 7 (23%) | 1.4 (0.6 – 3.1) | 0.49 |
| Active Malignancy | 5 (6.7%) | 4 (13%) | 1.8 (0.6 – 5.1) | 0.28 |
| Chronic skin disorder | 10 (13%) | 6 (19%) | 1.5 (0.6 – 3.6) | 0.40 |
| Immunosuppression | 7 (9.3%) | 7 (23%) | 2.3 (1.0 – 5.4) | **0.05** |
| Any chronic comorbidity | 26 (35%) | 24 (77%) | 4.8 (2.1 – 11.3) | **<0.001** |
| Home care | 1 (1%) | 9 (29%) | 6.4 (2.9 – 14.2) | **<0.001** |
| Ceiling of care | 2 (2.7%) | 6 (19%) | 3.0 (1.2 – 7.4) | **0.02** |
| Charlson comorbidity index | 2 (0.5-3.5) | 4 (2-5.5) | 1.15 (1.04 – 1.28) | **0.008** |

Table S1. Univariate associations between baseline characteristics and 30-day mortality. Hazard ratios, confidence intervals and p values come from Cox regression.

| Variable | Alive, N = 75 | Deceased,  N = 31 | HR (95% CI) | P value |
| --- | --- | --- | --- | --- |
| **Symptoms** |  |  |  |  |
| Fever | 46 (61%) | 14 (45%) | 0.5 (0.3 – 1.1) | 0.09 |
| Gastrointestinal | 36 (48%) | 16(52%) | 1.1 (0.5 – 2.2) | 0.79 |
| Localized pain | 27 (36%) | 10 (32%) | 0.9 (0.4 – 1.9) | 0.76 |
| Dyspnoea | 21 (28%) | 11 (36%) | 1.4 (0.7 – 2.9) | 0.86 |
| ENT symptoms | 8 (11%) | 2 (6.5%) | 0.6 (0.2 – 2.7) | 0.54 |
| Erythema | 14 (19%) | 5 (16%) | 0.8 (0.3 – 2.1) | 0.65 |
| Other symptoms | 10 (13%) | 3 (9.7%) | 0.7 (0.2 – 2.4) | 0.61 |
| **Disease severity** |  |  |  |  |
| Systolic BP, mmHg | 95 (80-111) | 103(85-130) | 1.01 (1.00 – 1.02) | 0.09 |
| Heart rate per minute | 110 (100-130) | 120 (100-130) | 1.00 (0.99 – 1.01) | 0.96 |
| Respiratory rate per minute | 30 (22-38) | 32 (30-40) | 1.04 (1.00 – 1.09) | 0.06 |
| Mental alteration | 10 (13%) | 12 (39%) | 2.7 (1.3 – 5.6) | **0.007** |
| NEWS score | 9 (6-11.5) | 11 (8-12) | 1.07 (0.97 – 1.19) | 0.17 |
| SOFA score | 9 (7-10) | 10 (7.5-12) | 1.11 (0.97 – 1.26) | 0.13 |
| Lactate, mmol/L | 5.2 (3.3-8.2) | 7.3 (4.3-10.4) | 1.11 (1.02 – 1.21) | **0.02** |
| Septic shock within 48 hours | 67 (89%) | 28 (90%) | 1.1 (0.3 – 3.6) | 0.90 |
| **STSS criteria** |  |  |  |  |
| Necrosis | 31 (41%) | 9 (29%) | 0.6 (0.3 – 1.4) | 0.25 |
| ARDS | 24 (32%) | 10 (32%) | 0.9 (0.4 – 2.0) | 0.85 |
| Rash | 22 (29%) | 2 (6.5%) | 0.2 (0.05 – 0.82) | **0.03** |
| Acute renal failure | 54 (72%) | 27 (81%) | 2.5 (0.9 – 7.0) | 0.09 |
| Coagulopathy | 44 (59%) | 19 (61%) | 1.1 (0.5 – 2.3) | 0.75 |
| Liver involvement | 39 (52%) | 21 (68%) | 1.7 (0.8 – 3.7) | 0.16 |

Table S2. Univariate associations between disease severity and 30-day mortality. Hazard ratios, confidence intervals and p values come from Cox regression.

| Variable | Alive, N = 75 | Deceased, N = 31 | HR (95% CI) | P value |
| --- | --- | --- | --- | --- |
| **Antibiotics** |  |  |  |  |
| Empiric betalactam |  |  |  |  |
| Benzylpenicillin | 1 (1.3%) | 2 (6.5%) | 2.8 (0.6-12.2) | 0.17 |
| Cloxacillin | 0 (0) | 1 (3.2%) | 4.4 (0.5-34) | 0.16 |
| Cefotaxime | 35 (47%) | 16 (52%) | Reference | NA |
| Piperacillin / Tazobactam | 8 (11%) | 8 (26%) | 1.8 (0.7-4.1) | 0.19 |
| Karbapenem | 31 (41%) | 4 (13%) | 0.34 (0.11-1.01) | 0.05 |
| Adjunctive Clindamycin | 67 (89%) | 25 (81%) | 0.53 (0.22 – 1.3) | 0.16 |
| **Other interventions** |  |  |  |  |
| Intensive or intermediate care unit | 72 (96%) | 27 (87%) | 0.38 (0.13 – 1.08) | 0.07 |
| Surgical intervention | 48 (64%) | 12 (39%) | 0.40 (0.19-0.82) | **0.01** |
| Mechanical ventilation or CPAP | 48 (64%) | 24 (77%) | 1.8 (0.8 – 4.1) | 0.19 |
| Renal replacement therapy | 25 (33%) | 10 (32%) | 0.92 (0.44-2.0) | 0.83 |
| Vasopressor therapy | 70 (93%) | 27 (87%) | 0.55 (0.19-1.6) | 0.26 |

Table S3. Univariate associations between treatment characteristics and 30-day mortality. Hazard ratios, confidence intervals and p values come from Cox regression.
